# Modulatory Effect of Association of Brain Stimulation by Light and Binaural Beats in Specific Brain Waves

**DOI:** 10.2174/1745017901713010134

**Published:** 2017-09-14

**Authors:** Mauricio Rocha Calomeni, Vernon Furtado da Silva, Bruna Brandão Velasques, Olavo Guimarães Feijó, Juliana Marques Bittencourt, Alair Pedro Ribeiro de Souza e Silva

**Affiliations:** 1 PhD Program in Mental Health, Federal University of Rio de Janeiro (IPUB/UFRJ), Rio de Janeiro, Brazil; 2 Sports Laboratory, State University of Rio de Janeiro (UERJ/RJ), Rio de Janeiro, Brazil; 3 PhD in Psychology, University Of Maryland, College Park, Brazil; 4 Brain Mapping Laboratory, Veiga de Almeida University (UVA/RJ), Rio de Janeiro, Brazil

**Keywords:** Brain Stimulation, Neuroplasticity, Brain Waves, EEG

## Abstract

**Introduction:**

One of the positive effects of brain stimulation is interhemispheric modulation as shown in some scientific studies. This study examined if a type of noninvasive stimulation using binaural beats with led-lights and sound would show different modulatory effects upon Alfa and SMR brain waves of elderlies and children with some disease types.

**Subjects:**

The sample included 75 individuals of both genders, being, randomly, divided in 6 groups. Groups were named elderly without dementia diagnosis (EWD), n=15, 76±8 years, elderly diagnosed with Parkinson’s disease (EDP), n=15, 72±7 years, elderly diagnosed with Alzheimer’s disease (EDA), n=15, 81±6 years. The other groups were named children with Autism (CA), n=10, 11±4 years, children with Intellectual Impairment (CII), n=10, 12 ±5 years and children with normal cognitive development (CND), n=10, 11±4 years.

**Instruments and procedure:**

Instruments were the Mini Mental State Examination Test (MMSE), EEG-Neurocomputer instrument for brain waves registration, brain stimulator, Digit Span Test and a Protocol for working memory training. Data collection followed a pre and post-conjugated stimulation version.

**Results:**

The results of the inferential statistics showed that the stimulation protocol had different effects on Alpha and SMR brain waves of the patients. Also, indicated gains in memory functions, for both, children and elderlies as related to gains in brain waves modulation.

**Conclusion:**

The results may receive and provide support to a range of studies examining brain modulation and synaptic plasticity. Also, it was emphasized in the results discussion that there was the possibility of the technique serving as an accessory instrument to alternative brain therapies.

## 1 INTRODUCTION

The functional brain activity normally occurs throughout high synaptic activity between large networks of neurons synchronously activated and producing rhythmic oscillations [1]. An important structure responsible for these oscillations is the thalamus, which has considerable influence on the cortex due to its relevance in the processing and retransmission of sensory and motor stimuli [2].

Due to this high processing activity the cortical neurons work at different frequencies depending on the voltage fluctuations from the ionic flow [3], producing rhythms denominated brain waves that are generated by the summation of electrical interactions of networked neurons, which can be measured in cycles per second or hertz [4]. Electrical interactions of neurons thus are related to states of consciousness [4, 5].

Thus, during the daily activities of a person, the cortex modulates the brain wave frequencies in the range of 0 to 40 Hz [6] to adapt to the demands of each behavior. In this way, brain waves occur naturally, independently of whether the person is in a state of rest or activity and it can be induced by external instruments [3] referred to as brain stimulators [7].

Noninvasive brain stimulation is a technique widely used in brain therapies and for other related medical and therapeutic objectives. The technique of flashing light, with parallel sound, in a binaural beats activation version has proofed to be effective in researches investigating brain functions improvement in a range of studies. For instance looking at gains of motor functions in a post stroke situation [8], temporal lobe activation of autistic children [9], attention concentration improvement of children diagnosed as low learners [6], working memory and attentional deficits of hyperactive children [10, 11], and for children kinesthetic sense improvement [12].

Thus, the research results above presented may give us linear evidence that noninvasive brain stimulation, used as above described, besides being a valuable instrument for research may also have a potential application for practical and clinical use in brain therapies. However, as in other types of brain stimulation use, this kind of technique is still very new, consequently demanding more scientific experimentation capable of indicating its use as an effective method. The present study is within that line of thought and designed to investigate if the common modulatory effect shown in studies as the above described would positively affect individuals of different ages and disease types.

## 2 MATERIALS AND PATIENTS

All diagnosed participants and the group of elderlies without dementia had regular examination to verify if there was any kind of evolution of their preexisting disease and/or the appearance of any sign of relevant imaging abnormality or other comorbidities. All of the above data were, registered, in an individual medical record updated every year and was available for research purposes. Due to the patient age differences, we had to obtain two different approvals from an ethics committee within the research area. This way, the procedures applied to the elderlies had approval from the ethics committee for human research of the Higher Education Institute of CENSA and received the number 983.807. The same Committee approved the procedures applied to the groups of children, being 296.193 the number to this second approval.

The sample included 75 individuals of both genders, who were representative of the following populations: elderly without dementia diagnosis (n=15) (EWD), elderly diagnosed with Parkinson’s disease (EDP) (n=15), elderly diagnosed with Alzheimer’s disease (EDA) (n=15), children with normal cognitive development (CND) (n=10), children with autism (CA) (n=10), and children with intellectual impairment (CII) (n=10).

The fifteen elderlies of the EWD group had a mean age of 76±8 years, and were admitted to reference shelters for elderly treatment in João Pessoa/PB city. They were included in this group based on the following criteria: lack of any symptoms of dementia indicated in the last individual medical record and confirmed by the nurses who attended them, scores higher than 80% in the Mini-Mental State Examination (MMSE), and regular participation in workshops and activities offered by the host institution in which they used to live. Any elderly who did not accept to be a volunteer, or for any reason, was unable to participate in the proposed assessment and/or in the planned intervention procedures, was excluded from the research.

To the EDP group, fifteen elderly with a clinical diagnosis of Parkinson made by a neurologist based on the symptoms and tests were included. Besides this exigence, in their last imaging evaluation were excluded any possibility of their having any other brain disease. The patients had a mean age of 72±7 years, and for insure the absence of dementia they had to obtain a score higher than 80% in the MMSE as well as the EWD group, and agreed to participate in the study. Elderly that, by any reason, was incapable to participate in the assessment and/or in the interventions procedure or had any type of dementia, associated with Parkinson’s disease, was excluded.

To compose the EDA group we had 15 patients from their host institution in João Pessoa/PB mean age of 81±6 years. The inclusion criterion was diagnosis of Alzheimer’s disease (DA) made by a neurologist who made the diagnostics based on the checklist of signals and symptoms and in a test of the National Institute of Neurological and Communicative Disorders and Stroke (NINCDS) and by their last image examination. All information regarding each patient examinations was registered in his/her medical control ?? and viable for research purposes. However, because the elderly were in different states of dementia, they also needed to obtain a minimum score of 40% in the MMSE. Elderly with any other associated comorbidity were also excluded. Any volunteer who, due to cognitive impairment, was unable to understand and participate in the proposed assessment and/or intervention was excluded.

The CDA group was composed of 10 children diagnosed with autism, of both genders and with a mean age of 11±4 years. As inclusion criterions, the children should have a recorded diagnosis of autism in a level of intensity influencing their daily common activities. All children who had high commitment enabling them to maintain a correct position of the stimulator and/or EEG electrodes were excluded.

The CII group had children from the same care centers in which the autistic patients were selected. The inclusion criterion was lower cognitive development than the chronological age but not associated with an autism condition. For this, after turning away the autism diagnosis, the children had their intelligence quotient (IQ) determinate by the center care and that information was individually recorded. Thus, 10 children (n=10) of both genders with a mean age of 12±5 years and with a moderate intellectual impairment were selected (IQ recorded lower than 55). Patients with a high level of commitment that prevented data collection were excluded (IQ lower than 25).

Finally, to CDA group, 10 children (n=10) of both genders and with a mean age of 11±4 years were selected. The inclusion criteria in the control group were cognitive development compatible with chronological age and any neurologic impairment that can indicate autism or intellectual impairment. Children whose legal guardians did not authorize participation or who clearly indicated their refusal to sign out the consent form were excluded.

The MMSE was applied in the EWD, EDA, and EDP groups [13], in which mental health was an inclusion criterion. However, the dependent variables observed in all groups were the alpha and SMR brain wave activities, which were monitored by using an electronic device known as Procomp Neurofeedback (Touch-Technology) [8, 9].

Another device used in all the experimental groups was the brain wave synthesizer (Sirius from The Mind Place Center in Canada). The device promotes noninvasive deep brain stimulation through the thalamus and is able to equalize the cerebral activity [7, 8, 11, 14, 15].

The present research aimed to verify the possible effects of mediation through stimulation of Alpha and SMR brain waves, on specific functions indicated by tests as deteriorated in the experimental groups. Some instruments, according to the experimental design, were utilized in certain groups but not in others. The digit span test [16] was used to evaluate the working memory of individuals in the EWD, EDP, and EDA groups and to verify the extent of memory with functional aspect. In the CA, CII, and CND groups, the specific instrument used was intended to induce mental states by the activation of mirror neurons responsive to the emotional states of happiness and sadness. For this purpose, pictures of real children made in moments of sadness (crying) and happiness (smile) were utilized, and one doll expressing sadness and the other expressing happiness were also utilized.

The first procedure was the protocol of brain stimulation by light and binaural beats. This had a total duration of 15 minutes, being applied individually in especially prepared rooms, in which dispersers and distractors, such as temperature and external sounds, were controlled. The total time of stimulation was divided such that a specific frequency band was stimulated for 3 min. In other words, the 0-to-3-minute waves were induced at 8 Hz, the 4-to-6-minute waves at 10Hz, the 7-to-9-minute waves at 12Hz, the 10-to-12-minute waves at 14 Hz, and the 13-to-15-minute waves at 15 Hz; this guaranteed that all spectra of alpha and SMR brain waves were covered. Another procedure that was common to all groups was the monitoring of alpha and SMR brain waves, which was done at two different moments: firstly, before the brain stimulation protocols by light and binaural beats and the training of a specific impaired function in each experimental group; and secondly, after these moments. However, this monitoring was variable in application according to the brain area associated with each functional variable to be observed.

In the EWD, EDP, and EDA groups, in which functional impairment was associated with memory problems, the electrode attachment points were F7, A1, and A2, defined according to the International 10-20 System. Point F7 relates to visual and auditory working memory [17], whereas points A1 and A2 places were used as references to the obtaining data. The elderlies in these groups were individually conducted to the prepared room for the data collection procedure. As a common instruction, during this procedure they were all asked to seat for the electrodes placement. All the placement points had to be sanitized with 70% alcohol and even a conductive past to facilitate signal capture was used. After this procedure, the alpha and SMR brain waves were recorded for a period of 2 minutes, during which the individuals remained seated in silence, with minimal movements.

In the CA, CII, and CND groups, to study the processing of emotional stimuli, the alpha and SMR brain waves were observed in the temporal lobe, the location of the amygdala, which is related to response to emotional stimuli, independent of whether these were pleasant or unpleasant [18]. Thus, the electrodes were placed at point T6 due to its relation to Wernicke's area [19]. Furthermore, the localization of the electrodes in the right hemisphere is favorable because several studies have shown that the right hemisphere is more important than the left hemisphere in the recognition of stimuli demanding emotional responses. Points A1 and A2 were also used as reference points. The monitoring in these groups was carried out for 6 minutes, with the children exposed to a different emotional stimulus for 2 minutes each. That is, in the first 2 minutes, a neutral stimulus was presented, with the children remaining seated and without contact with any image referring to any emotions; in the next 2 minutes, images of other children expressing feelings of happiness were presented, and in the last 2 minutes, the children were exposed to images referring to feelings of sadness.

In the following, having presented the procedures applied commonly to all groups, we describe those that were carried out specifically in the EWD, EDP, and EDA groups. The previously selected participants in these groups had their mental health evaluated based on the MMSE to determine whether they met the inclusion criteria for each group. The evaluation was done individually in the same room in which data collection was carried out with the use of an electroencephalogram. The MMSE evaluation was done 7 days before beginning of the other records, because this time was necessary to those results which were analyzed to maintain the stringency of inclusion criteria.

Another procedure that was applied specifically to the EWD, EDP, and EDA groups was the evaluation of working memory. This was done after the mental health evaluation and before the recording of alpha and SMR brain wave activities. The digit span test was also applied individually and immediately after the initial recording of brain wave activity. The elderly were asked to repeat a sequence of numbers in correct order and immediately after dictation, with the objective of observing the memory space. The test results were expressed as perceptual values of the digit sequence repeated in correct order. This same procedure was repeated after the period of working memory training described below.

The working memory was trained in the EDP and EDA groups, which consisted of elderly with this function committed. The training protocol was applied during 10 sessions carried out on alternate days, for a total of 20 days of intervention. The training was always done at the end of the brain stimulation by light and binaural beats, for a total time of approximately 15 minutes.

Specific procedures were also applied in the CA, CII, and CND groups. In these groups, the functional variable of interest was the reactivity of mirror neurons in the cortical area related to emotional processing. To understand this objective, after the initial monitoring of brain waves, the children in these groups received brain stimulation by light and binaural beats after having their processing of emotional states trained. This training lasted for a period of 4 minutes, with 2 minutes dedicated to the processing of images depicting states of happiness and the remaining 2 minutes devoted to the processing of images expressing states of sadness. Four figures were shown to each child in the same order: two depicting happiness and two expressing sadness. As a training form, during the first minute of exposition, the children were asked to identify the feeling expressed in each image; and in the next minute, the children were allowed to interact freely and spontaneously. Soon after, the monitoring of brain waves was repeated, applying the same criteria described above.

It is important to note that in these last groups, due to the difficulty of displacement of participants to place in training and evaluation, it was decided to observe a possible acute effect of proposal stimulation. Such difficulty was not encountered with the participants in the first three groups, which consisted of elderly admitted to the host institution.

## 3 STATISTICAL ANALYSIS

To determine which is the best variable to represent the central tendency of groups and the most indicated inferential test to comparisons into the groups was applied the Shapiro Wilk normality test. This test indicated samples between 30 and 50 individuals and allows the classification of the data as parametric or non-parametric. Because the data were nonparametric, descriptive analysis was applied, with the median as a measure of central tendency, and the extreme values and standard deviation as measures of group dispersion. Intragroup inferences were obtained by using the signal test. For the differences into the groups to be considered statistically significant, the p value should be lower than 0.05 in the signal test.

## 4 RESULTS

Table **1** shows the data on the central tendency, extreme values, and dispersion of the groups of elderlies without dementia (EWD), elderlies with a diagnosis of Alzheimer’s disease (EDA), and elderlies with a diagnosis of Parkinson’s disease (EDP). The data were collected before and after the intervention procedure for each group.

The same data of Table **1** were plotted in Fig. (**1**), showing more easily the increase in central tendency for the averaged activity observed for all instances of the Alpha waves, except for elderly group with Parkinson's diagnosis. It also illustrates that the coefficient of variation for same brain waves suffered reduction in most of the groups, being stable only for the elderly with Parkinson's diagnosis but increasing in the elderly with Alzheimer's diagnosis.

As shown in Fig. (**1**), for alpha brain waves, the association between the stimulation by light and binaural beats and the training of impaired function induced both quantitative and qualitative effects on the activity of neuron networks associated with the task. These findings appear to be associated with better results in the digit span test considering that all groups achieved better results after the methodological intervention, as it can be observed in Fig. (**2**).

Continuing the descriptive analysis of the data, we can observe Table **2** in which are presented the results of the descriptive analysis of data related to the EEG recordings made in the CND, CII, and CA groups.

From Table **2**, we can see that the average activity of Alpha and SMR waves was higher in the post test than in the pre-test except for SMR waves of children with Autism. However, when we observe the coefficient of variation, we can note that there was reduction in the alpha waves in most of the groups, with exception in the one linked to children with autism. However, when we observe the coefficient of variation of SMR waves, we can note another pattern. In most of the groups, there was increase in the coefficient of variation, being the exception the group of children with intellectual impairment in which a small reduction was registered. In Fig. (**3**), we can observe the average activity of SMR wave between pre-test and post-test registered in all groups.

However, to meet the objectives of this research, it is necessary to test the hypothesis regarding the effectiveness of modulation of brain activity by light and binaural stimulation and to carry out an inferential analysis of the pre- and post-intervention scores of each group.

Table **3** shows the p-values for the comparison between the pre- and post-intervention scores. As indicated in the table, only the recorded differences between the average alpha brain wave activities in the EDA group and the coefficient of variation of this same brain wave in the CA group were statistically significant. However, in this last group, the difference between the average activities of the alpha brain wave showed a strong tendency toward statistical significance.

A more in-depth analysis indicated that in the EDA group, the brain stimulation by light and binaural beats produced a 97% increase in the average activity of the alpha waves. In the CA group, the increase of 13% in the average activity of alpha brain waves approached statistical significance, whereas the increase of 114% in the coefficient of variation was statistically significant. Despite the descriptive difference registered in the other groups, the inferential analysis does not show any statistically significant difference.

## 5 DISCUSSION

The descriptive data shown in Tables **1** and **2** clearly indicate some effect of stimulation by light and binaural beats on the Alpha and SMR brain wave activities in all the observed groups. Except in the EDP group, as shown in Table **1**, an increase in the average alpha brain wave activity was observed, which may be related to the increased activity of neurons in the brain area monitored by the EEG. At the same time, the coefficient of variation of this same brain wave was decreased in four of the six focal groups analyzed.

Calomeni *et al.* [8] observed a similar phenomenon, which was associated with a significant functional increase in the motor task deteriorated by stroke. Recently, Silva Vernon *et al.* [9] concluded that such changes occurred due to the modulation of synaptic activity as a result of stimulation by light and binaural beats. Thus, based in other findings, Silva Vernon *et al.* [7] classified stimulation by light and binaural beats as deep stimulation once its modulatory action occurs through the brainstem and thalamus structures.

According to Palva and Palva [20], alpha waves have large amplitude and occur during moderate levels of brain activity, playing a crucial role in the construction of neuron networks [3]. Besides, mainly in the elderly, the alpha brain wave activity can improve the recognition of words and facilitate the working memory (Desai *et al.* [3]). The results related to Alpha waves were consistent with findings of Pennisi *et al.* [21], Bella *et al.* [22] and Pennisi et al [23] which also showed a cortical hyperexcitability in the Alpha frequency produced by Transcranial Magnetic Stimulation (TMS) in individuals with vascular dementia, vascular cognitive impairment-no dementia and Alzheimer’s disease.

Another group that evidenced different pattern was the CA group because the results of the average activity and coefficient of variation of Alpha waves may seem inconclusive. However, in the case of autistic individuals, which, according to Lapenta [24], have significantly lower brain excitability as compared to control individuals. These findings show an increase in brain excitability, which may indicate possible neuroplastic changes, at least temporarily [9].

In relation to the average activity of SMR brain waves, the data show that only in the CA group there was no increase in this activity. Besides, none defined pattern in the coefficient of variation was observed: some groups showed an increase, whereas others showed a decrease. This could be attributed to the physiology of this brain wave, which, when recorded in the motor cortex, is related to a state of readiness for movement. When recorded in any other region of the brain, this brain wave became a beta wave; occuring only when it is extremely necessary and is disabled as soon as the task ends [25].

In conclusion, brain stimulation by light and binaural beats showed different levels of effectiveness in the modulation of alpha and SMR brain wave activities, with varying effects according to the characteristics of the sample and the site of the EEG recording. In this study, modulation of the activity of the alpha wave was found to be more effective than to the SMR waves. It is noteworthy here that the observed effects in this present study are of acute nature; thus, the differences could be more significant if a methodology including more training sessions associated with brain stimulation was used. However, our results are enough to suggest with some confidence that the technique can serve as a basis for brain therapies looking for neuroplastic and behavioral restructuring, at least, for individuals with the same characteristics as those in the sample of this study.

## Figures and Tables

**Fig. (1):**
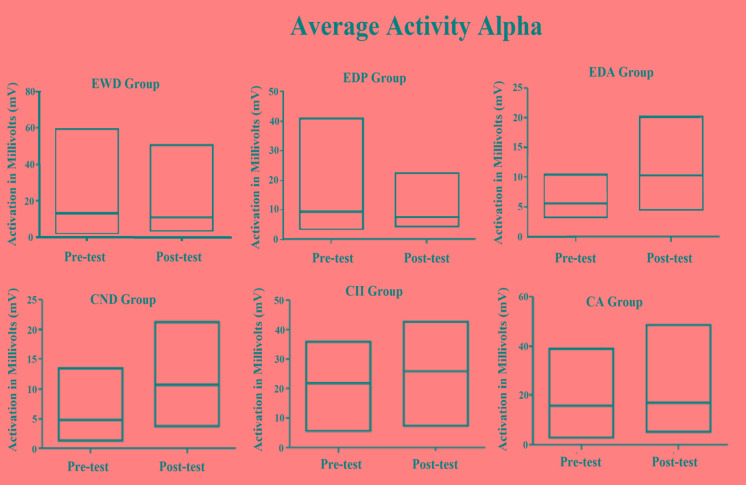
Central tendency data and extremes related to brain wave activity Alfa in pre and post intervention times in each study group.

**Fig. (2):**
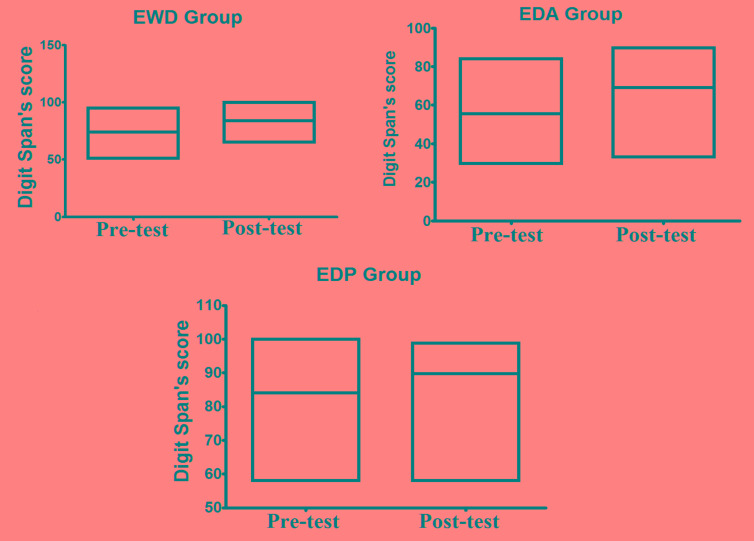
Results of pre-test and post-test obtained through Digit Span Test applied to evaluate the working memory function in the EWD, EDA and EDP groups.

**Fig. (3):**
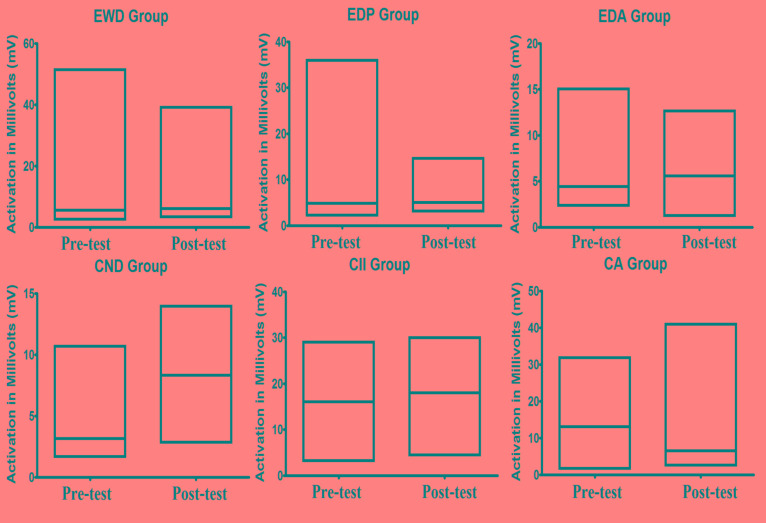
Central tendency data and extremes related to brain wave activity SMR in pre and post intervention times in each study group.

**Table 1:** Description of central tendency data, dispersion and extremes of groups EWD, EDA and EDP, collected pre and post intervention.

Elderly Group Without Dementia Diagnosis (EWD)													
	Alpha Waves					SMR Waves					Wok Memory Task		
Average Activity		Coefficient of Variation			Average Activity		Coefficient of Variation			Digit Span		
Pre intervention	Post intervention	Pre intervention		Post intervention	Pre intervention	Post intervention	Pre intervention		Post intervention	Pre intervention		Post intervention
Minimum	2.18	3.25	0.03		0.03	2.52	3.31	0.03		0.04	51		65
Maximum	59.77	50.81	1.29		2.56	51.44	39.2	1.17		1.72	95		100
Median	6.29	7.06	1.05		0.69	5.6	6.05	0.65		0.5	74		84
Standard deviation	15.56	12.07	0.41		0.66	12.91	8.94	0.39		0.49	14.46		9.96
Group of Elderly with Alzheimer's Diagnosis (EDA)													
	Pre intervention	Post intervention		Pre intervention	Post intervention	Pre intervention	Post intervention		Pre intervention	Post intervention	Pre intervention		Post intervention
Minimum	3.09	4.53		0.1	0.08	2.36	1.25		0.08	0.1	29.54		32.95
Maximum	10.55	20.23		1.64	1.84	15.03	12.64		1.47	1.58	84.09		89.77
Median	4.49	8.84		0.88	0.78	4.44	5.59		0.44	0.54	56		67
Standard deviation	2.59	4.46		0.48	0.45	3.21	2.81		0.44	0.40	16.58		17.34
Group Elderly people with Parkinson's Diagnosis (EDP)													
	Pre intervention	Post intervention		Pre intervention	Post intervention	Pre intervention	Post intervention		Pre intervention	Post intervention		Pre intervention	Post intervention
Minimum	2.99	3.99		0.03	0.04	2.15	3.09		0.04	0.04		57.9	57.95
Maximum	41.03	22.5		1.64	3.4	36.01	14.68		1.8	1.31		100	98.86
Median	6.8	5.99		0.48	0.49	4.82	5.01		0.49	0.43		84	90
Standard deviation	9.253	4.56		0.383	0.8	8.27	3.06		0.49	0.30		15.32	15.77

**Table 2:** Description of central tendency data, dispersion and extremes of groups CND, CII and CA, collected pre and post intervention.

Children with Cognitive Development Normal (CND)								
	Alpha Waves				SMR Waves			
Average Activity		Coefficient of Variation		Average Activity		Coefficient of Variation	
Pre Intervention	Post Intervention	Pre Intervention	Post Intervention	Pre Intervention	Post Intervention	Pre Intervention	Post Intervention
Minimum	1.31	3.68	0.48	1.29	1.67	2.82	0.35	1.13
Maximum	13.54	21.26	11.36	4.56	10.68	13.95	9.43	5.81
Median	3.67	10.02	2.78	1.39	3.16	8.33	1.01	1.27
Standard Deviation	4.13	6.66	3.74	1.18	3.45	3.60	3.05	1.61
Children with Intellectual Impairment (CII)								
	Alpha Waves				SMR Waves			
	Average Activity		Coefficient of Variation		Average Activity		Coefficient of Variation	
	Pre Intervention	Post Intervention	Pre Intervention	Post Intervention	Pre Intervention	Post Intervention	Pre Intervention	Post Intervention
Minimum	5.47	7.35	0.41	0.38	3.13	4.43	0.35	0.38
Maximum	36.13	42.85	0.63	0.5	29.01	30.05	0.63	0.79
Median	21.31	24.69	0.46	0.43	16.04	17.99	0.46	0.44
Standard Deviation	9.21	13.59	0.072	0.042	8.98	9.81	0.09	0.11
Children with Autism Diagnosis (CA)								
	Alpha waves				SMR waves			
	Average Activity		Coefficient of Variation		Average Activity		Coefficient of Variation	
	Pre Intervention	Post Intervention	Pre Intervention	Post Intervention	Pre Intervention	Post Intervention	Pre Intervention	Post Intervention
Minimum	2.83	5.06	0.39	0.44	1.66	2.57	0.30	0.43
Maximum	39.18	48.74	0.88	1.48	31.80	40.92	0.83	1.33
Median	7.49	8.49	0.49	1.05	13.02	6.57	0.46	1.03
Standard Deviation	16.00	16.20	0.18	0.33	12.40	14.56	0.20	0.32

**Table 3:** p values for the comparisons between the scores of the average activity and coefficient of variation of brain waves alpha and SMR in pre- and post-intervention.

	Signal test Pre-intervention x Post intervention					
	Alpha Brain Waves					
	EDP	EDA	EWD	CA	CII	CND
p (Binomial) =	0.1509	0.0005*	0.3953	0.0547	0.3633	0.2266
Power of test =	0.8491	0.999	0.6054	0.9431	0.6382	0.7752
	SMR Brain Waves					
	EDP	EDA	EWD	CA	CII	CND
p (Binomial) =	0.1509	0.5	0.3953	0.6563	0.1133	0.3633
Power of test =	0.8491	0.5	0.6054	0.6585	0.8861	0.6382
	Coefficient of Variation of Alfa Brain Waves					
	EDP	EDA	EWD	CA	CII	CND
p (Binomial) =	0.3036	0.6047	0.3953	0.0107*	0.2266	0.2266
Power of test =	0.6972	0.6054	0.6054	0.9866	0.7752	0.7752
	Coefficient of Variation of SMR Brain Waves					
	EDP	EDA	EWD	CA	CII	CND
p (Binomial) =	0.3953	0.5	0.0898	0.1094	0.2744	0.3633
Power of test =	0.6054	0.5	0.9093	0.8897	0.7268	0.6382

* p<0,05
